# Clinical associate students’ perceptions of factors that influence their developing professional identity: a qualitative study

**DOI:** 10.1186/s12909-023-04109-3

**Published:** 2023-02-21

**Authors:** Aviwe Mgobozi, Lakshini McNamee, Ian Couper

**Affiliations:** 1 Division of Clinical Associates, Department of Family Medicine and Primary Care, University of Witwatersrand, Johannesburg, South Africa; 2 Centre of Health Professions Education, Stellenbosch University, Stellenbosch, South Africa; 3 Department of Health Science Education, University of Cape Town, Cape Town, South Africa; 4 Department of Global Health, Ukwanda Centre for Rural Health, Stellenbosch University, Stellenbosch, South Africa

**Keywords:** Clinical Associate, Physician Assistant, Clinical Officer, Mid-level health care worker, Professional Identity, Professional Identity formation

## Abstract

**Background:**

New cadres of clinicians, known as clinical associates, physician assistants, or clinical officers have evolved globally within many health systems to broaden access to care by increasing human resources. The training of clinical associates started in 2009 in South Africa, entailing the attainment of knowledge, clinical skills, and attitude competencies. Less formal educational attention has been focused on the process of developing personal and professional identities.

**Method:**

This study utilized a qualitative interpretivist approach to explore professional identity development. A convenient sample of 42 clinical associate students at the University of Witwatersrand in Johannesburg were interviewed using focus groups to explore their perceptions of factors that influenced their professional identity formation. A semi-structured interview guide was used in six focus group discussions, involving 22 first-year and 20 third-year students. The transcriptions from the focus group audio recordings were thematically analyzed.

**Results:**

The multi-dimensional and complex factors that were identified were organized into three overarching themes, identified as individual factors which derive from personal needs and aspirations, training-related factors consisting of influences from the academic platforms, and lastly, student perceptions of the collective identity of the clinical associate profession influenced their developing professional identity.

**Conclusion:**

The newness of the identity of the profession in South Africa has contributed to dissonance in student identities. The study recognizes an opportunity for strengthening the identity of the clinical associate profession in South Africa through improving educational platforms to limit barriers to identity development and effectively enhancing the role and integration of the profession in the healthcare system. This can be achieved by increasing stakeholder advocacy, communities of practice, inter-professional education, and the visibility of role models.

**Supplementary Information:**

The online version contains supplementary material available at 10.1186/s12909-023-04109-3.

## Background

Since 2009, the University of the Witwatersrand in South Africa has been training Clinical Associates (ClinAs), a profession that was introduced into the South African healthcare system in 2004 to increase the accessibility of healthcare providers to the South African population [1, 2]. The founding concept drew on the physician assistant in the USA and the clinical officer in Tanzania, aiming to alleviate the burden of work on doctors and address the shortage of primary healthcare clinicians [3–5]. The ClinA is trained within a biomedical curriculum to work in collaboration with the healthcare team, with the supervision of a medical practitioner [5]. The curriculum design is based on the integration of theory and practice, training students to take patient histories, perform physical examinations, formulate diagnoses, perform diagnostic and therapeutic procedures and manage patients, as per scope of practice [6].

Some challenges faced by ClinA professionals in South Africa include limited governmental support in job creation, uncertain career pathing, lack of supervision, and wavering policy planning [7, 8]. These challenges directly and indirectly affect undergraduate ClinA students and their future aspirations. Some students struggle with the novel nature of the programme, and students often have a lack of knowledge about the ClinA programme before enrolment. The roles and responsibilities of the profession are often unclear to students, peers, and families, with some students seeking to bypass practising as ClinAs and to proceed into a training programme to become medical doctors. Brown et al. identified similar issues amongst physician associate students in the UK [9].

The Carnegie Foundation for the Advancement of Teaching called for reforms to improve the preparation of 21st-century healthcare professionals. Amongst other things, their report recommends that medical education formally support the shaping of professional identity [10].

This research study utilises the definition by Cruess and Cruess [11] for professional identity which states a manifestation of one’s identity that expresses the norms, values and behaviours of the profession of choice. A strengthened professional identity results in individuals embodying values, attributes, and characteristics that are required to positively influence their clinical practice. This in turn results in better clinical outcomes [12–14].

The inability to integrate personal and professional identities results in identity dissonance. The tern, identity dissonance defines a disconnection between how an individual envisions themselves against the expectations in line with the characteristics of the new professional identity. This causes internal conflict. Joseph et al. [15] suggests that identity dissonance is an emotional state that results in students doubting their self-worth and questioning their values and ambitions due to the incompatibility of their personal and professional identity. Personal identities are influenced by the manner individuals perceive themselves (ego) and how they are seen and socially grouped by society (social identities). The effect of medical history and hierarchies within society on professional identity, is an avenue for exploration as suggested [16]. Professional identity dissonance is suggested to yield consequences of student discomfort, feeling disconcerted, and perceived unpleasantness of the professional role [17]. On the contrary, having a clear and established professional identity positively influences the individual’s decisions and professional demeanour, and results in effective clinical practice. There is an increasing body of literature on professional identity that suggests a strengthened professional identity nurtures a positive disposition and leads to good clinical outcomes [17, 18].

There is limited literature that explores the influences that contribute to the professional identity of Clinical Associates and related professionals, and the beneficial or detrimental influences on the students’ developing professional identities. This study explored ClinA students' perceptions of factors that influenced their professional identity formation.

## Materials and methods

The research study is a qualitative, cross-sectional study that used an interpretive paradigm. Focus group discussions were utilised to collect participant lived experiences and detailed beliefs of their worldviews. Interpretivism is most suitable for the study as it allows for the subjectivity of the data and recognizes the existence of multiple realities. The research was conducted in the Faculty of Health Sciences, University of the Witwatersrand, in Johannesburg, South Africa in 2018.

### Data collection

The principal investigator sent an email communication to all ClinA students in 1^st^ year (52 students) and 3^rd^ year (30 students) to share the study details and invite students to participate in the study. Students voluntarily responded and signed up to participate. There were no exclusion criteria and probability convenience sampling was used.

Focus group discussions were ideal for this study as it enabled students to respond to shared experiences, engage and react to each other thus creating an in-depth, enriched discussion. Munday re-enforces the method as she recommends the usage of focus groups to explore collective identities [19]. Focus group discussions were organised and scheduled by the principal investigator. The participants signed written consent forms before the start of the discussions. All focus group discussions were guided by a semi-structured interview format, audio-recorded, transcribed verbatim and thematically analysed. The principal researcher created the interview questions in line with the research questions and objectives. 42 participants were divided into six groups. The groups consisted of 22 first-year students and 20 third-year students. Each focus group discussion varied in duration, lasting from 45 min to 1 h 45 min.

The focus group discussions were held in pre-booked campus venues. They were facilitated by the principal investigator (AM) with a senior researcher (MM, DP & FK) and research assistant (NS) present at each focus group discussion to formulate observation notes. A team discussion was held at the end of each focus group discussion to reflect on the sessions.

### Data analysis

The principal researcher (AM) and the research assistant (NS) manually transcribed the audio recordings. The principal researcher (AM) confirmed the transcripts by re-listening to the audio recordings and interpreted the data using the interpretative paradigm. Factors that influence professional identity were identified by the principal researcher (AM). The data was iteratively and inductively analysed using the thematic analysis method as described by [20]. MAXQDA (VERBI Software, 2016) was used for manual coding and the categorization of codes to construct categories of themes and recurring units of meaning that could later be identified as the factors that influence professional identities. Investigator triangulation was used to increase the credibility of the findings by comparing the findings from the audio recording, field notes and observations by the researchers (AM) (MM), (FK), and (DP).

The principal researcher discussed the findings from the focus group discussion with the senior researchers (IC) (LM). This enabled an in depth analysis of the data due to the contribution made from the comparison of interpretation [21].

## Results

The demographic details of the 42 participants are presented in Table 1.

**Table 1:** Demographics of the study participants

Focus Group	Year of Study	No. of Participants	Females	Males	Age Range (Years)
1	1^st^ year	8	6	2	18–21
2	3^rd^ year	7	5	2	20–22
3	3^rd^ year	5	4	1	20–23
4	3^rd^ year	8	5	3	20–22
5	1^st^ year	7	4	3	20–23
6	1^st^ year	7	4	3	19–30

The results were categorized into three themes representing the factors that were reported to influence the ClinA students’ developing professional identity, namely, individual factors, training-related factors and perceptions of identity.

### Theme one: Individual factors

The first theme entails student perceptions of factors relating to their personal selves that had influenced their developing professional identity, with personal desires as the initial influences shaping the developing professional identity. The sub-themes in this category include the influence of unfulfilled personal needs, students’ core aspirations, and intrinsic and extrinsic motivation.

The perception of unfulfilled personal needs resonated in both year groups. Students expressed concern and unease regarding the disconnect of initial aspirations when enrolling in the ClinA programme and abandoning their initial career goals that were accompanied by prospects of attaining social status and financial security.

*“do I see myself as a clinical associate: I don’t know, the thought of being a clinical associate scares me because I almost feel like if I graduate and kinda become a clinical associate I’ve almost failed myself and failed what my ultimate dream was because I ultimately want to be a paediatrician and kind of specialise in children.” (3rd year student – FGD 4)*

*“At the end of the day you still need to meet your own needs as a person. You need salary, you need to do things. We are still very young.” (3rd-year student – FGD 2)*

This shows that student desires have a prominent influence on their sense of professional identity. In addition, these student desires had an enabling or limiting effect on their motivation. Internal and external motivations emerged in all student focus groups as defining stimuli to pursue becoming a clinical associate. Students who were intrinsically motivated saw value in the profession through their interaction with patients.

*“You must be self -aware, know yourself. Know what you are capable of and know where you’re going in life because then that will give you confidence to be a clinical associate, even with what everybody is saying about you or about your profession. It’s believing [in] yourself, having confidence in what you are doing, because at the end of the day, I know that we are looked down upon and everything but what we do in hospital, that advocates for us.” (1st year student – FGD 1)*

*“…the passion is still there, if there is anything I can say, and I won’t flinch after saying this is that I am proud to be a clinical associate, I have worked with consultants, MOs and I have done so much to stand up for myself.” (3rd year student – FGD 3)*

A third-year student appreciated positive encouragement from healthcare workers, which helped increase their motivation, saying that *"the staff opinion about us in second and third year also helps you grow to a level where you end up having to be forced to be competent and independent in your own way".(3rd-year student – FGD 2)*

Enrolling in a programme that has similar educational objectives and a similar scope of practice to that of medical doctor training contributed to student confusion regarding the clinical associate professional identity. The unclear demarcations in the scope of practice seemed to prod students towards the initial aspiration of many of them to pursue becoming doctors where they felt the value of the profession was more established.

*“You don’t see the difference and the ‘value-added’ component to us clinical associates. I think that’s one thing that really stood out for me during my entire training, which is that if I’m already doing this, why don’t I just, okay, the sentiment that continues to reverberate itself is that since you are already doing this, why don’t you just do medicine?” (3rd-year student – FGD 3)*

### Theme two: Training related factors

The second main theme covers training-related factors encountered in the educational environment, namely the medical school campus and the healthcare facilities that formed the clinical training platform. Students raised positive and negative factors that were seen to influence their developing professional identities.

Positive factors that shaped students’ developing professional identity were noted as a result of the experiential learning that occurs on the clinical training platform. These factors entailed early patient exposure, patient contact, and affirming medical preceptors. The early patient contact was highlighted by most students as a remarkable and positive experience that helped them better understand the value of being a clinical associate.

*“But when you see the difference you make in someone else’s life and knowing that no, that was my knowledge. I put that in my head, I actually went, studied that skill. You know what I mean? It makes you feel that actually now, even though I might not do medicine, but as a clinical associate I am competent enough where I am supposed to be. So I feel for me, seeing it and practising it myself brought the positive factors.” (3rd year student – FGD 2)*

Concurrent to the positive experience was frustration that related to the differently structured academic programme. The nature of the integrated curriculum was perceived by some students as a limitation due to differences in design from other faculty programmes. ClinA students perceived judgment from other students within the faculty of health sciences who enrol in traditional programmes structured in modular forms.

*“People look down on you just because you don’t have separate modules. I mean, we do go to the lab, we do see cadavers, we do anatomy and physiology, but our peers themselves, they judge our competence according to the kind of modules that we do.” (1st year student – FGD2)*

In addition to their perceptions of the training programme, students expressed concerns regarding inter-programme student socialization and its effects on their developing professional identities. From this experience, an existing institutional culture of student hierarchy and segregation amongst health science students was raised.

*“We rank each other. Like without even saying it, but we know that okay, you’re a nurse, your place is here, you’re a clinical associate, your place is here, oh you’re studying medicine? Okay, your place is here.” (1st year student – FGD 1)*

The combination of programme structures and early professional socialization affects the development of students’ identities.

### Theme three: Perceptions of identity

Students expressed a strong need to understand their profession’s identity. They felt the profession was poorly understood in the South African society and healthcare system. Students sought to find defining concepts for the identity of the clinical associate profession.

First-year students demonstrated uncertainty about their identity based on the lack of understanding of the profession's identity and nomenclature.

*“I was still not clear what exactly a clinical associate is, and with this degree, what am I going to be called? Am I going to be called a doctor?” (1st year student – FGD 1)*

*“I think we focus too much on what we do instead of who we are, it’s more like what is a clinical associate? Well, we can do X, Y, Z but like what are we? I can’t even really answer that question, to be honest, but I mean, nobody defines a nurse from what they do. They define them from like who they are.” (1st year student – FGD 2)*

The doctor identity was commonly imposed on students by other healthcare workers. An example given was of a third-year student who mentioned an encounter during ward rounds where the doctor said to a patient "my young doctor here is going to see you". In this case, the perception was created for patients to see the clinical associate students incorrectly as student doctors despite some effort to correct this by students.

*“… so that’s where the whole profession and identity gets lost because now even in hospital, there are some clinical associates who are like, you know what, I have introduced myself so many times as a clinical associate to doctors but they refuse to address you as a clinical associate.” (3rd year student – FGD 2)*

In their quest to understand the markers that define the profession’s identity, students raised concerns about the profession’s scope of practice being unclear. The supervision requirement of qualified clinical associates also altered how students see their capabilities. The understanding of the supervision required instilled an inferiority complex and a sense of performance inadequacy. Most students felt a sense of mistrust would develop from patients, colleagues and society.

*“Factors that sort of have an impact on our identity as we are said to be, we are defined as ‘supervised’, the term ‘supervised’. In our training we are so ‘joined to the hip’ of the doctor that our sense of independence sort of does not exist. So the minute you have to see a patient yourself, you doubt your competency.” (3rd year student – FGD 2)*

Largely, third-year students experienced marginalization and a lack of recognition from hospital medical staff, which led to a sense of professional isolation, questioning of belonging, and being undermined. Participating in doctor- and nurse-led clinical teams challenged students' ideas of where clinical associates fit in as they felt continuously overlooked and undermined. The negotiation of identities prevails amongst third-year students as they struggle with a lack of role models and, by default, are required to learn about their roles from different professionals. Disparaging remarks and negative commentary by senior doctor preceptors influenced students' views of themselves as clinical associate students.

*"When you do a simple mistake, they'll be like that clinical associate did this and this and this. You're like, the thing is, you know the profession, but you just won't be recognized in a way. It's like whether I'm good or bad, refer to me as a clinical associate, because now people are like if everything bad that happens, it's because of the clinical associate, then there is going to be that rep[utation] of oh, the clinical associate did it. But what about the good things? They'll be like oh, the doctor did it." (3rd year student – FGD 2)*

*“… we interact with them as doctors do, and because of that, it kind of creates, centres our identity around a doctor, and because our identity is kind of centred around what doctors are, we have no clear identity(.)we have no clear identity because we are constantly striving for, we are constantly holding onto what they are.” (3rd year student- FGD 3)*

In addition, the career progression in the country and labour-related issues faced by working clinical associates within the public sector affected students, namely unclear career progression strategies relating to postgraduate opportunities and professional advancement.

*“Is there a clear path for growth in the clinical associate degree” (3rd year student – FGD)*

*"… if the same thing would have to happen as clinical associate, that there is some sort of hierarchy that will recognize the fact that I have been in the clinical setting for five years, or I have worked in the obs and gynae department for five years, can I have some sort of recognition of the amount of work that I have put in for the past five years, and be differentiated from a graduate." (3rd year student – FGD 3)*

Lastly, suggestions were made of possible solutions to assist identity formation, namely increasing role models in the training platforms and increasing awareness of the profession.

*"I need someone who can be a father figure or something to me, because I am a clinical associate, I'm not a doctor. I need to know how to practice within my field, not just copying and pasting from other people. Like oh, nurses put up catheters, I want to learn how to do that, oh doctors do surgeries, let me tag there, oh, OTs do chest physio, you know. You need something that's going to guide you, like okay, this is how to go about being a clinical associate." (3rd year student – FGD 4)*

## Discussion

The study affirms that professional identity formation is a multi-dimensional, relational and contextual process that involves the influence of ego identity, personal identity, and social identity [22]. The students’ perceptions of their selves’, their future projections of their selves and their understanding of hierarchies and medical field norms, influences their experience of professional identity formation.The personal and socio-cultural influences on professional identity formation are deeply influenced by the socialization that occurs prior to enrolment, at institutional level, and at the clinical environments where hierarchy and professional silos are still dominant in the South African healthcare system.

The students’ developing professional identities are initially influenced by their internal dispositions, career aspirations, and pursuit of security and success. An early identity dissonance manifests at the enrolment phase of the programme where training to be a ClinA did not always correlate with initial career ambitions. It appears in the senior student responses that, while many gradually mature into a sense of ownership of, comfort in and identification with their chosen profession, several ClinA students move through the programme without achieving a measure of satisfaction with their career choice due to their envisioned future professional selves not being aligned with their current professional journey and developing identity, as they instead continue to aspire to become medical doctors. This sense of having unmet needs such as fulfilment of personal aspirations, experiencing societal validation, and financial needs directly affects motivation, as theorized by Maslow [23]. This also correlates with the study findings by Brown et al. [9] amongst physician associate students in the United Kingdom, who saw the physician associate training programme as unfulfilling and as a path to becoming a medical doctor.

Our findings suggest that positive encouragement and ongoing motivation resulted in feelings of professional acknowledgment, inclusivity and recognition. This affirmation was highlighted to positively influence professional identity formation amongst physician associates in England [9]. Students with a positive outlook were driven by intrinsic motivation arising from personal enjoyment of clinical tasks, patient interactions, and an appreciation of achieving clinical competence which enabled them to see their contribution to patient care.

The impact of the (early) patient exposure encountered in the clinical rotations positively influences the students’ developing professional identity. The value of this practical component of the programme was persistently emphasized by the clinical associate students in the study. Similarly, patient encounters by medical students have a positive effect in strengthening their professional identity [24, 25]. By their third academic year, the students in our study were more appreciative of the responsibility of providing healthcare and acknowledged the graduate competencies and exit outcomes of the programme. This demonstrated a developing professional identity.

Role modelling has been identified as a key influencer on professional identity development [9, 26–28]. It is an important driver of the hidden curriculum. The Hidden curriculum comprises of implicit influences such as the environmental culture, rituals, norms and contextual forces, not part of the formal curricula that affects the students during their work place based learning [29].

The scarcity of qualified ClinAs working in the training health facilities results in a lack of role model visibility. This causes young ClinA students to assimilate the roles of their present preceptors, who are mainly medical doctors. Helmich et al. [30] suggests the placement of health science students within a discipline other than their profession has benefits for learning but may perpetuate identity dissonance. Kaiser [31] proposes that an individual’s professional identity is constructed through the recognition of differing roles between professions which demarcates the defining aspects of identity. As the students learn skills and professional attitudes from the doctors, nurses and allied healthcare professionals, the hidden curriculum influences the professional identity formation as the students organically emulate receptor behaviours. This may cause internal conflict in understanding what roles to internalize as clinical associate students.

Current professional challenges faced by qualified ClinAs in South Africa influence the undergraduate students as they realize the complexities and politics of the working world. Our findings show that a lack of opportunity for professional development contributed to poor professional esteem amongst students, as found by Hao et al. [32]. in their study of Chinese nursing students. These similarities emphasize the need for academic institutions and governmental policy makers to work synergistically and intentionally to implement formal practices that effectively shape new and diverse health professional identities.

This study has highlighted the altering negotiation of identities amongst clinical associate students. The perceived ill-defined identity and positionality of the clinical associate profession in South Africa largely influences students’ developing identity. This in turn influences students’ dispositions, their agency and their efforts to assimilate their personal identities to establish a professional identity. There is a need to unravel both enabling and inhibitory effects of the hidden curriculum on professional identity and seek ways opportunities to improve ClinA education in order to strengthen identify formation. An opportunity arises for exploration of how the historical and the socio-cultural impact of the current South African healthcare system influences the professionalization of new cadres.

### Limitations of the study

The study was conducted in a single institution, and findings cannot be generalized to clinical associate students at other universities in South Africa, though anecdotally it accords with their experiences. The lack of gender diversity in the focus groups is due to the nature of the student intake in the ClinA program at the university with women predominating in the course. This echoes the gender groupings found in a Wits study that implements graduate tracking [33].

### Reflexivity

The principal investigator is a clinical associate graduate from the 2009 cohort and was a lecturer during the data collection period. AM practiced personal reflexivity by formulating notes consisting of personal feelings and reactions. AM kept a journal to record the reaction to the multiple truths and the realizations of the unregulated hidden curriculum that followed the focus group discussions.

## Conclusion

In order to effectively incorporate healthcare professionals into existing healthcare systems, a critical exploration of the enablers to the development of professional identity is required for new graduates not only to attain sufficient knowledge, skill competency, and professionalism but also to develop a strengthened and integrated professional identity. A multi-directional, solutions based approach is required (Table 2).

**Table 2:** Summary of Recommendations

Factors influencing Students developing professional identity	Recommendation
Internal disposition	Increasing education and awareness of the profession to assist individuals to make well informed decisions regarding enrolment. This is an attempt to decrease potential identity dissonance
Programme Enrolment	Reviewing admission criteria to enrol applicants who meet the programme minimum requirements and possess early manifestations of the dispositions required in the clinical associate health care professional
Intrinsic and extrinsic motivation	Increasing visibility of clinical associate role models
Clinical Training experiences	Exploring explicit pedagogical practices which support the formation of professional identity. E.g • Fostering early patient exposure • Inter-professional education • Role modelling • Building communities of practice
Profession related challenges: Regulations and policy governing the clinical associate profession	Advocacy for the profession is required at all levels of the health care system. Synergy is necessary between academic institutions and governmental structures to strengthen policies to contribute to the
The identity of the profession in the country	Increasing marketing of the profession to raise awareness of the profession

Pedagogical strategies can be explored to support the development of student professional identities [17, 27]. Inter-professional education, active role modelling and building communities of practice are amongst some identified interventions for strengthening professional identity [34–37].

Continued advocacy for the ClinA profession at local and national levels in South Africa is of paramount importance. The perception of the profession by students and society is one that is influenced by individual and collective experiences and understanding of the role of the Clinical Associate. The developing identity of the profession in South Africa has a critical bearing on students’ professional identities. This highlights the need for continued monitoring and evaluation of the role, impact, support and identity of ClinA professionals within the South African healthcare system.

## Supplementary Information

**Additional file 1.**

## Data Availability

The datasets and/or analysed during the current study are available from the corresponding author on reasonable request.
