# Supplementary material for: Clinical associate students’ perceptions of factors that influence their developing professional identity: a qualitative study
Source: BMC Med Educ. 2023 Feb 21;23:125. doi: 10.1186/s12909-023-04109-3 (PMC9945349; doi:10.1186/s12909-023-04109-3)
Supplement: Supplementary file 1 — Additional file 1. [file 12909_2023_4109_MOESM1_ESM.pdf]

Clinical Associate students' perceptions of factors that influence their developing professional identity: A Qualitative Study

*Aviwe Mgobozi[1, 2], Lakshini Mc Namee[3], Prof Ian Couper

Semi-structured interview guide: First Year Students

1. How did you feel when you found out you were accepted into the Clinical Associate programme?
2. Do you see yourself as a practicing Clinical Associate? What does this mean to you?
   - Probing Question: Tell me more about your reason.
3. How do you relate to the Clinical Associate profession?
4. What has helped you understand what it means to be a Clinical Associate?
   - Probing Question: Tell me more about the negative and positive factors
5. What do you think you need personally to improve your understanding of the profession? What will be most helpful to you?

Semi Structured Interview guide: Third Year Students

1. How did you feel when you found out you were accepted into the Clinical Associate programme?
2. Do you see yourself as a practicing Clinical Associate? What does this mean to you?
3. How do you feel your understanding of being a clinical associate has changed since first year?
4. What does the process of becoming a Clinical Associate mean to you?
   - Probing Question: Do you see yourself as a Clinical Associate?
   - Probing Question: How do you relate to the Clinical Associate profession?
5. Have you identified any factors which helped you better understand yourself as a Clinical Associate?

Probing Question: Participants can comment on positive influences and negative influences which have challenged their understanding of themselves as a Clinical associate student.

Ensuring Interaction

The researcher will allow the focus group discussion to continue naturally and use the following stimulants to encourage discussion.

a. Probing questions have been indented under the main interview questions. They will be used to further gain deeper understanding into participant responses.

b. The researcher will recap participant answers to gain confirmation and to reflect on what has been. Participants may remember to provide additional missed points.

c. The researcher will try balance the conversation and chance the power dynamics in the group from encouraging introverted participants to have their chance to speak.
